# Value of intraoperative pathological diagnosis in decision-making regarding resection of well-differentiated retropharyngeal liposarcoma: A case report

**DOI:** 10.1016/j.ijscr.2021.106466

**Published:** 2021-10-04

**Authors:** Masakazu Ikeda, Satoshi Fujii, Yohei Morishita, Ryuichi Hayashi

**Affiliations:** a Department of Head and Neck Surgery, National Cancer Center Hospital East, Kashiwa, Japan; b Department of Otolaryngology, Fukushima Medical University, Fukushima, Japan; c Division of Pathology, National Cancer Center Exploratory Oncology Research & Clinical Trial Center, Kashiwa, Japan; d Department of Molecular Pathology, Yokohama City University Graduate School of Medicine, Yokohama, Japan

**Keywords:** Liposarcoma, Head and neck cancer, Retropharyngeal space

## Abstract

**Introduction and importance:**

Preoperative diagnosis of well-differentiated liposarcoma (WDLS) in the retropharyngeal space is challenging because of the difficulty in obtaining a biopsy tissue specimen that will yield the microscopic findings necessary for a definitive pathological diagnosis. This report describes a case of retropharyngeal WDLS that was successfully diagnosed intraoperatively, which allowed radical resection.

**Case presentation:**

The patient was a 60-year-old man suspected of having a lipomatous tumor in the retropharyngeal space. On imaging, the tumor contained linear septum-like structures that were prominent behind the larynx. Pathological examination was performed using fine-needle aspiration cytology and core needle biopsy specimens. However, no malignant features were found. Given that partial biopsy of the retropharyngeal tumor by puncture was anatomically limited, we decided to collect appropriate tissue specimens for intraoperative pathological examination. During the operation, we biopsied the tumor, including the hard portion behind the larynx, anticipating inclusion of the septum-like structures seen on imaging. A pathological diagnosis of WDLS was successfully made and the tumor was completely excised.

**Clinical discussion:**

The fibrous septum with induration is important for intraoperative diagnosis of WDLS. The fibrous septum within the tumor was palpable as a rubbery hard portion.

**Conclusion:**

WDLS in the retropharyngeal space could be successfully resected surgically by making a rapid intraoperative pathological diagnosis using appropriately selected tissue sampled from a hard portion of the tumor.

## Highlights

• Preoperative pathological diagnosis of well-differentiated liposarcoma in the retropharyngeal space is challenging.
• Pathological diagnosis of well-differentiated liposarcoma requires tissue sampling including the fibrous septum within the tumor.
• Well-differentiated liposarcoma could be diagnosed by intraoperative pathological examination to select the area containing the fibrous septum.

## 1 Introduction

Well-differentiated liposarcoma (WDLS) is defined by the World Health Organization as an atypical lipomatous tumor in the category of liposarcoma [1] and is treated mainly by surgery. Although WDLS has a low potential for metastasis and a good prognosis, incomplete resection may result in local recurrence [2], which can lead to dedifferentiation and a less favorable outcome. Therefore, if preoperative diagnosis is difficult. Intraoperative diagnosis will be important for radical resection of WDLS with an adequate surgical margin.

Pathological diagnosis of tumors located in deep spaces within the body, such as the retropharyngeal space, is usually performed using specimens obtained by fine-needle aspiration (FNA) cytology or core needle biopsy (CNB) [3]. However, it is difficult to collect a tissue specimen of WDLS in the retropharyngeal space by puncture because it is surrounded by vital structures. To date, there has been no report of a tissue collection method that enabled intraoperative pathological diagnosis of WDLS. Herein, we report a case of WDLS in the retropharyngeal space that was diagnosed by intraoperative pathological examination using an appropriate tissue specimen.

This case has been reported in line with SCARE criteria [4].

## 2 Presentation of case

**Fig. 1:**
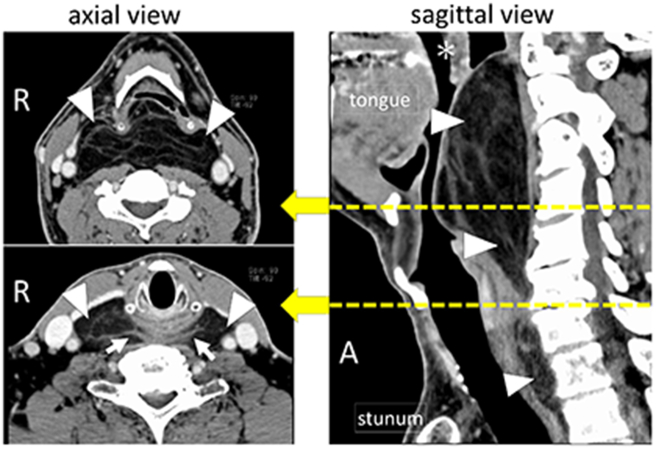
Computed tomography scanning. Axial view: The tumor in the retropharyngeal space (arrowhead). The septum-like structures were prominent behind larynx (arrow). Asterisks(*):uvula. Sagittal view: The tumor of retropharyngeal space was extended from soft plate to mediastinum (arrowhead).

**Fig. 2:**
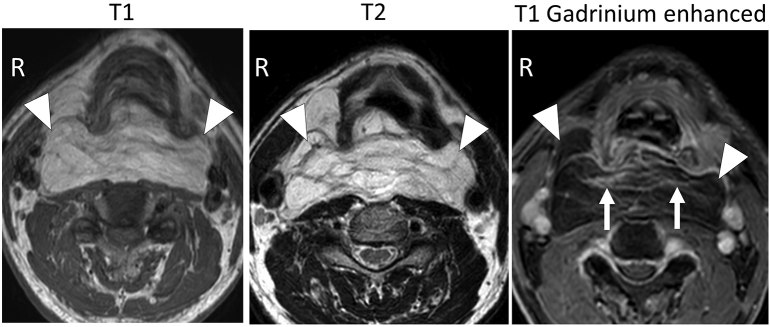
Magnetic resonance imaging. The tumor showed high intensity in T1 and T2 weighted images (arrowhead). Septum-like structures in the tumor were enhanced with Gadrinium (arrow).

The patient provided written consent for publication of this case report. A 60-year-old man visited our hospital with a chief complaint of retropharyngeal swelling. He had been treated for obstructive sleep apnea for 3 years at a local clinic but had started to experience symptoms of dyspnea. A computed tomography scan revealed a low-density area in the retropharyngeal space indicating a tumor. The tumor extended from the soft palate to the superior mediastinum and contained linear septum-like structures that were prominent behind the larynx (Fig. 1). On imaging, the tumor showed high intensity on T1-weighted and T2-weighted gadolinium-enhanced magnetic resonance images, suggesting a lipomatous tumor with septum-like structures (Fig. 2). Tissue for pathological examination was obtained by FNA cytology and CNB. No malignancy was detected in the cytology specimen. The CNB specimen consisted of mature adipose tissue with no malignant features. Given that our ability to obtain a partial biopsy of the retropharyngeal tumor by puncture was anatomically limited, we decided to collect tissue specimens more appropriate for intraoperative pathological examination.

In the operation, the tumor was first resected from the area where it was not adherent to the surrounding organs. The overall texture of the tumor was soft and there was a partially hard inner portion. We biopsied the tumor, including the hard portion, anticipating inclusion of the area of septal fibrosis. The tumor was successfully and pathologically diagnosed intraoperatively as WDLS. The tumor was attached to the pharyngeal constrictor and anterior vertebral muscles, which were also excised as surgical margin. The tumor was completely excised while preserving the larynx.

**Fig. 3:**
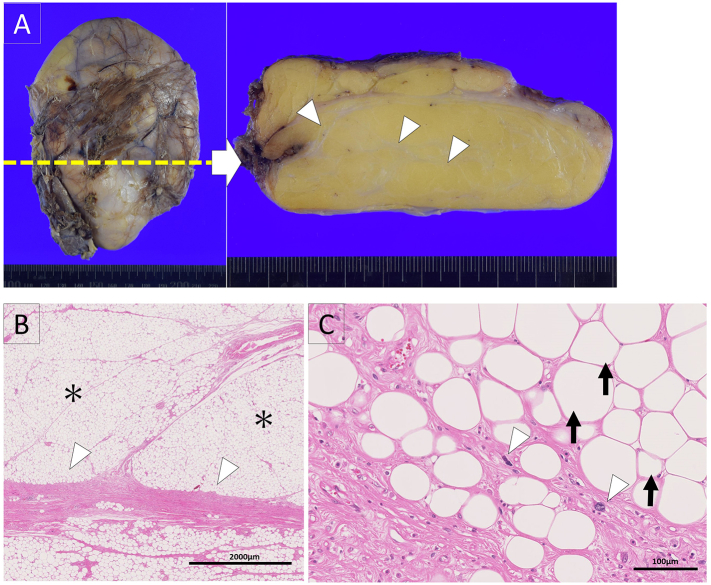
A: Macroscopic finding of the tumor. The tumor was occupied by yellowish adipose tissue with white septum-like structures (arrow). B: Microscopic finding of Hematoxylin and eosin staining. The tumor was composed of mature adipose tissue(*) with fibrous septum-like structures (arrowhead). Bar:2000 μm. C: High power view of hematoxylin and eosin staining. The adipocytes varied in size (arrow). The atypical stromal cells with nuclear atypia presented in the septum-like structures(arrowhead). Bar: 100 μm.

Most of the tumor was occupied by yellowish adipose tissue with a white fibrous septum-like structure (Fig. 3-A). Microscopically, most of the tumor was composed of mature adipocytic tissue with a fibrous septum (Fig. 3-B). The adipose cells varied in size, unlike in benign lipoma. The fibrous septum included atypical stromal cells with nuclear atypia, such as hyperchromatic or multiple nuclei (Fig. 3-C).

The patient remains alive without recurrence at 36 months after surgery.

## 3 Discussion

WDLS was diagnosed in this case by intraoperative pathological examination. Only five cases of WDLS in the retropharyngeal space have been reported, none of which were pathologically diagnosed as WDLS at the time of surgery [5], [6]. Tissue biopsy for a tumor in the retropharyngeal space is difficult to perform using a puncture technique, such as FNA and CNB, because of the risk of puncture-related damage to the surrounding structures. Moreover, it is difficult to differentiate WDLS from benign lipoma by microscopic observation of a small tissue biopsy specimen because both tumors contain many areas composed of mature adipocytes. Therefore, a pathological diagnosis of WDLS in the retropharyngeal space is difficult to confirm by FNA or CNB. Moreover, an open biopsy of sarcoma carries a risk of dissemination of the tumor [7].

Correct sampling of the tumor tissue is important for histopathological diagnosis by intraoperative pathological examination because most WDLS lesions are composed of mature adipocytic tissue. The microscopic findings when a pathological diagnosis of WDLS is made include atypical stromal cells with enlarged and hyperchromatic nuclei [8]. In the present case, the atypical stromal cells were in the fibrous septum-like structure within the tumor; this area was palpable as a hard portion inside a soft tumor (Fig. 3C). Pathological diagnosis of WDLS requires tissue sampling; in our case, this included the fibrous septum-like structure within the tumor. The area containing the histological element can be selected based on macroscopic findings through visual observation. The pathological findings of WDLS include adipocytes of variable size and appearance of atypical stromal cells and lipoblasts. Atypical stromal cells are regarded as pathologically important findings in the diagnosis of WDLS and tend to be present in the fibrous septal area in these tumors.

On imaging, WDLS usually presents as a largely lipomatous mass with non-lipomatous components, such as a fibrous septum. The finding of a thick fibrous septum (>2 mm) on gadolinium-enhanced magnetic resonance images can accurately differentiate WDLS from benign lipoma [9]. However, the appearance of a benign lipoma may be more complex than that of a WDLS, in which case the difference cannot be distinguished radiologically [10]. Imaging studies in our case showed that the fibrous septum of the tumor was dense at the level of the larynx (Fig. 1, Fig. 2). In that area, the fibrous septum was palpable as a rubbery hard portion that was extending into the soft portion of the tumor. Therefore, it is important for surgeons to select the area containing the histological element and submit it for intraoperative pathological diagnosis. Awareness of this report may help to improve the prognosis of WDLS.

## 4 Conclusion

WDLS that develops in the retropharyngeal space can be successfully resected surgically by making a rapid intraoperative pathological diagnosis using appropriately selected tissue sampled from a hard portion of the tumor.

## Funding

This research did not receive any specific grant from funding agencies in the public, commercial, or not-for-profit sectors.

## Ethical approval

This report was conducted in compliance with ethical standards. Informed written consent has been obtained and all identifying information is omitted.

## Informed consent

Written informed consent was obtained from the patient for publication of this case report. A copy of the written consent is available for review by the Editor-in-Chief of this journal on request.

## Research registration number

Not applicable.

## Guarantor

Masakazu Ikeda, Satoshi Fujii.

## Provenance and peer review

Not commissioned, externally peer-reviewed.

## CRediT authorship contribution statement

Ikeda M: Management of case, conception of study, acquisition of data, analysis and interpretation of data, drafting the article, and revision of article.

Fujii S: Revision of article, and final approval of the version to be submitted.

Morishita Y: Acquisition of data.

Hayashi R: Management of case and approval of the final version for submission.

## Declaration of competing interest

The authors have no competing interests to declare.
